# The efficacy of prophylactic prochlorperazine injections at the initiation of opioid injections in preventing opioid-induced nausea and vomiting among patients with end-stage cancer

**DOI:** 10.20407/fmj.2022-034

**Published:** 2023-08-28

**Authors:** Kazuki Imai, Akihiko Futamura, Miyo Murai, Akihiro Ito, Norimasa Tsuzuki, Masanobu Usui

**Affiliations:** 1 Fujita Health University Hospital, Toyoake, Aichi, Japan; 2 Department of Surgery and Palliative Medicine, Fujita Health University, School of Medicine, Toyoake, Aichi, Japan; 3 Department of Pharmacy, Fujita Health University Nanakuri Memorial Hospital, Tsu, Mie, Japan

**Keywords:** OINV, Morphine, Oxycodone, Prochlorperazine injection

## Abstract

**Objectives:**

Antiemetics have been widely recommended for treating opioid-induced nausea and vomiting (OINV). According to a previous study, the use of prophylactic prochlorperazine at the initiation of treatment with oral oxycodone was ineffective in preventing OINV. This study examined whether prochlorperazine injection prevents OINV and induces drowsiness in patients with end-stage cancer (a different patient population from the previous study).

**Methods:**

Patients with end-stage cancer who received opioid injections for more than 5 days between April 2017 and March 2020 were classified into two groups: the opioid and prochlorperazine injection group and opioid alone group. Their systemic conditions were evaluated on the basis of the performance status and the palliative performance scale, a prognostic indicator.

**Results:**

Of 325 patients who received opioid treatment during the study period, 156 patients met the inclusion criteria. Of these, 103 patients and 53 patients were classified into the opioid and prochlorperazine injection group (prochlorperazine) and opioid alone groups (placebo) , respectively. There was no significant difference in characteristics, age, gender, performance status, or palliative performance scale results between the 2 groups. OINV developed in 4 patients in the opioid and prochlorperazine injection groups and in 1 patient in the opioid alone group. Given that sleep disturbance develops in many patients with end-stage cancer who had a specific condition, it is difficult to conclude regarding the relationship between prochlorperazine injection and drowsiness, although this study examined this relationship.

**Conclusions:**

As with the previous study, prophylactic prochlorperazine injection was ineffective in preventing OINV in patients who received opioid injections.

## Introduction

Pain develops in 20%–50% of cancer patients,^1^ and moderate to severe pain develops in approximately 80% of patients with advanced cancer.^2^ Pharmacotherapy with opioids is the mainstay of standard treatment in these patients. The World Health Organization 3-step analgesic ladder recommends the use of non-opioid and opioid analgesics according to the pain intensity.^3^

Opioid-induced nausea and vomiting (OINV) is a side effect of opioid analgesics, remarkably decreasing patient’s quality of life. Thus, the prevention of this condition is extremely important. Nausea develops in approximately 20% of patients and vomiting in approximately 10% after the initiation of oral or patch opioids.^4^ Several studies have suggested that the use of antiemetics and opioid switching, including from oral administration to injection and use of an alternative opioid with different mechanisms of action, improve OINV symptoms.^5–11^

However, no clear criteria regarding prophylactic treatment for preventing OINV are described in the guidelines, such as the National Comprehensive Cancer Network,^12^ the Multinational Association of Supportive Care in Cancer, and the European Society for Medical Oncology.^13^

Tsukuura et al.^14^ have reported that the use of prophylactic prochlorperazine at the initiation of treatment with oral oxycodone is ineffective in preventing OINV.

Of the 120 patients included in the previous study,^14^ 92 patients (76.7%) had an Eastern Cooperative Oncology Group performance status (PS) of ≤2. Patients with end-stage cancer experience decreased appetite, generalized weakness, nausea, and decreased level of consciousness due to cachexia progression. Such conditions make oral administration difficult,^15^ resulting in the use of injection. Unlike in the previous study,^14^ PS is estimated to be ≥3 in many patients with end-stage cancer. Therefore. this study examined their systemic conditions based on the palliative performance scale (PPS),^16^ a prognosis indicator used in the palliative care, along with the Eastern Cooperative Oncology Group PS. A previous study^14^ has reported that prochlorperazine is ineffective in preventing OINV; however, drowsiness, a side effect, is frequently found. Thus, the present study evaluated whether the incidence of drowsiness differs according to cancer stages. Patients in this study were classified into two groups: those who received both opioid and prochlorperazine injections, and those who received opioid injections alone. The primary end point was the antiemetic efficacy of prochlorperazine injection. The secondary end point was the incidence of drowsiness after prochlorperazine injections.

## Methods

### Patients

We included 325 patients with end-stage cancer who were hospitalized in the palliative care ward of Fujita Health University Nanakuri Memorial Hospital between April 2017 and March 2020. The patients were classified into two groups: those who received prochlorperazine injection 5 mg as an antiemetic for five days after the initiation of opioid injections (prochlorperazine group) and those who received opioid injections alone (placebo group). We retrospectively examined their medical records.

### Evaluation method

**Figure 1:**
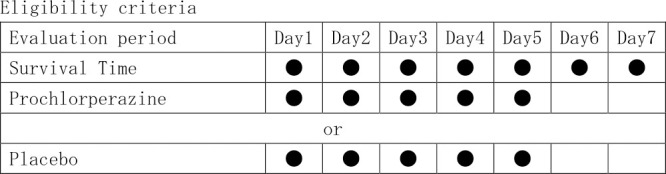
Eligibility criteria

The inclusion criteria, as shown in Figure 1, were patients who were alive for longer than seven days after the initiation of opioid injections. Patients in the placebo group received opioid injections for five consecutive days, and patients in the prochlorperazine group received prochlorperazine injections for five consecutive days.

Nausea and vomiting were determined to be present if additional administration of prochlorperazine injections or other antiemetics were required.

The exclusion criteria were as follows:

1. Patients who did not meet the inclusion criteria, 2. Patients who experienced vomiting before opioid injection, 3. Patients who used other antiemetics (haloperidol, metoclopramide, octreotide, chlorpheniramine) and steroids, 4. Patients who were unable to communicate, 5. Patients with brain metastasis, 6. Patients with hypercalcemia.

### Statistical analysis

Mann–Whitney U test and Fisher’s t-test were used to compare quantitative and qualitative data, respectively, between the two groups. The significance level was set at P<0.05. The odds ratio was used to examine the differences in the incidence of nausea and vomiting between the two groups and between men and women. Statistical analyses were performed with the use of Statcel 3 (4 Steps Excel-Statistics, OMS Publishing Ltd, Saitama, Japan).

### Ethical considerations

This study was approved by the ethics board of Fujita Health University (approval number: CI20-641) and performed in compliance with the “Guidelines on Medical Research for People.”

## Results

A total of 325 patients started receiving opioid injections during the study period. Of these, 169 patients who did not meet the inclusion criteria were excluded; accordingly, 156 patients were included in this study.

The detailed breakdown of the excluded patients was as follows. Twenty patients had died before their evaluation was completed. Twenty-five patients experienced nausea before opioid injections. Fifty-six patients received haloperidol injections for treating delirium. Thirty-nine patients received antiemetics other than prochlorperazine injections, and of these, eight patients received octreotide injections for treating peritoneal dissemination or gastrointestinal obstruction. Fourteen patients were unable to communicate. Ten patients did not complete a treatment regimen with opioid or prochlorperazine injections during the study period. Three patients had received opioid injections since they were hospitalized and. One patient had brain metastasis and another had hypercalcemia, which were factors contributing to nausea and vomiting.

**Figure 2:**
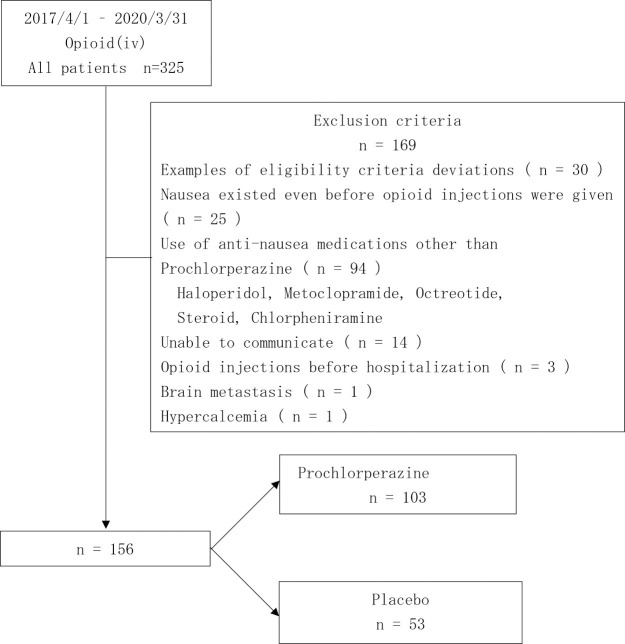
Screening of the study patients

Of the156 patients included, 103 patients (66.0%) and 53 patients (34.0%) were assigned to the prochlorperazine and placebo groups, respectively (Figure 2).

**Figure 3:**
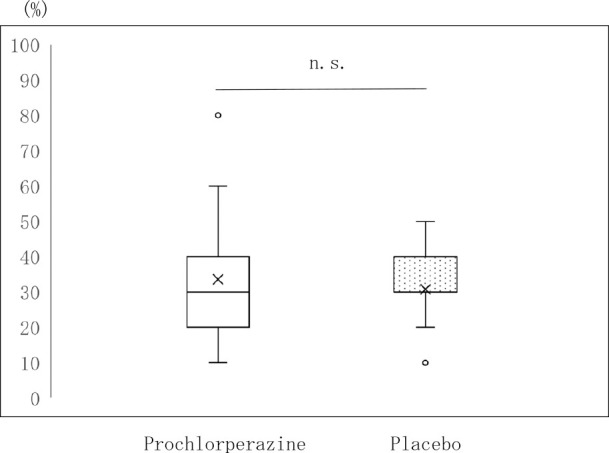
Palliative Performance Scale

There was no significant difference in gender, age, or Eastern Cooperative Oncology Group PS results between the two groups. Furthermore, no significant difference was observed in cancer types between the two groups (Table 1). No significant difference was found in the PPS results between the two groups (Figure 3).

The opioid types of both the groups were examined before and after opioid switching (Table 2).

In the prochlorperazine group (103 patients) before opioid switching, the most commonly used opioids were oral oxycodone in 28 patients (27.2%), followed by oral morphine in 15 patients (14.6%), fentanyl patch in 8 patients (7.8%), and oral hydromorphone in 1 patient (1.0%). There were 51 patients (49.5%) with no history of opioid treatment (opioid-naïve patients).

In the prochlorperazine group after opioid switching, the most commonly used opioid injections were morphine injections in 69 patients (67.0%), followed by oxycodone injections in 32 patients (31.1%), and hydromorphone injections in 2 patients (1.9%).

In terms of opioid switching among the 15 patients who used oral morphine, 14 patients (93.3%) and 1 patient (6.7%) started receiving morphine and oxycodone injections, respectively. Among the 28 patients who used oral oxycodone, 16 patients (60.7%), 11 patients (39.3%), and 1 patient (3.6%) started receiving morphine injections, oxycodone injections, and hydromorphone injections, respectively. The one patient who used oral hydromorphone started receiving oxycodone injections. Among the eight patients who used fentanyl patch, five patients (62.5%) and three patients (37.5%) started receiving morphine and oxycodone injections, respectively. Among the 51 opioid-naïve patients, 34 patients (66.7%), 16 patients (31.4%), and 1 patient (2.0%) started receiving morphine injections, oxycodone injections, and hydromorphone injections, respectively.

In the placebo group (53 patients) before opioid switching, the most commonly used opioids were oral oxycodone in 24 patients (45.3%), followed by oral morphine in 9 patients (17.0%), fentanyl patch in 8 patients (15.9%), and oral hydromorphone in 2 patients (3.8%). Ten patients (18.9%) were opioid-naïve patients.

In the placebo group after opioid switching, the most commonly used opioid injections were morphine and oxycodone injections in 26 patients for each (49.1%), followed by hydromorphone injections in 1 patient (1.8%).

In terms of opioid switching among the nine patients who used oral morphine, all patients started receiving morphine injections. Among the 24 patients who used oral oxycodone, 6 patients (25.0%) and 18 patients (75.0%) started receiving morphine and oxycodone injections, respectively. Among the two patients who used oral hydromorphone, one patient started receiving oxycodone injections and the other with hydromorphone injections. Among the eight patients who used fentanyl patch, five patients (62.5%) and three patients (37.5%) started receiving morphine and oxycodone injections, respectively. Among the ten opioid-naïve patients, six patients (60.0%) and four patients (40.0%) started receiving morphine and oxycodone injections, respectively (Table 2).

There were 51 (49.5%) and 10 (18.9%) opioid-naïve patients in the prochlorperazine and placebo groups, respectively (p=0.0002) (Table 3). During the first five days after the initiation of opioid injections, OINV developed in 4 of the 103 patients (3.9%) and 1 of the 53 patients (1.9%) in the prochlorperazine and placebo groups, respectively, with no significant difference between the two groups (p=0.502) (Table 4).

Drowsiness developed in 4 patients in the prochlorperazine group and 12 patients in the placebo group. Among the four patients in the prochlorperazine group, one patient (25.0%) used a sleeping drug and its dose was increased. Among the 12 patients (66.7%) in the placebo group, 6 patients used a sleeping drug, and 2 of them increased opioid doses.

Thirteen patients in the prochlorperazine group and eight patients in the placebo group did not experience drowsiness even after using hypnotic drugs, such as sleeping pills and antidepressants. Of these patients, nine patients (69.2%) in the prochlorperazine group and two patients (25.0%) in the placebo group complained of insomnia, resulting in the administration of hypnotic drugs.

After excluding patients who had a factor contributing to drowsiness, the incidence of drowsiness that was considered to be induced by prochlorperazine injection was compared between the two groups. The results showed no significant difference (P=0.193) (Table 5).

## Discussion

Although this study included patients with end-stage cancer (i.e., a different patient population from a previous study) and used a different dosage form of prochlorperazine, it suggested that prochlorperazine injection was ineffective in preventing OINV in these patients.

The most commonly used opioids before opioid switching were oral oxycodone both in the prochlorperazine and placebo groups. After opioid switching, the use of morphine injection was increased. Many cancer patients with advanced stages experience distress, such as pain, dyspnea, and malaise because they have difficulty receiving oral administration. Physicians tend to use morphine injections for treating such patients based on the basis of their clinical experiences. By taking this tendency into account, we believed that the percentage of opioid switching and that of opioid-naïve patients who started receiving opioids for preventing OINV was higher in the prochlorperazine group than in the placebo group (Table 3). Furthermore, when the incidence of OINV among opioid-naïve patients was evaluated (Table 4), the odds ratio did not show the superiority of the prochlorperazine group over the placebo group.

The present study showed that the incidence of OINV was higher in the prochlorperazine group than in the placebo group. The results of the present study were inconsistent with those of the previous studies.^10,11^ These studies reported that prochlorperazine may be effective in preventing nausea and vomiting, although they did not focus on prophylactic prochlorperazine in preventing OINV.

The incidence of nausea and vomiting was 3.2% (five patients) when the prochlorperazine and placebo groups were combined. The rate was lower than the result of a previous study^4^ that used oral or patch opioids. There are two possible explanations for this difference: First, the acquisition of drug resistance, which was reported by Stephenson et al.^17^ Second, opioid switching from oral opioid to opioid injection and introduction, which were reported by Enting et al.^7^ Opioid tolerance may develop in the patients in this study. Furthermore, they used opioid injections, which are associated with fewer incidences of nausea and vomiting than oral or patch opioids.

The incidence of drowsiness after prochlorperazine injection was examined as the secondary end point. The results showed no relationship between them, which were inconsistent with the results reported by Tsukura et al.^14^ Such differences between the two studies may be explained by differences in the patient population. A previous study^18,19^ has shown that 23%–70% of patients with end-stage cancer (i.e., study population of the present study) experience drowsiness.

This study indicated that prophylactic prochlorperazine injection was ineffective in preventing OINV and drowsiness in patients with end-stage cancer, with no significant difference between the prochlorperazine and placebo groups. Such results suggest that uniform rules for antiemetic prescription are not required for patients who start to receive opioids. Given that factors contributing to nausea and vomiting vary, such as gender, cancer stages, and central and/or peripheral nervous systems, appropriate use of antiemetics according to these factors and patients’ symptoms is desired.

This study has several limitations. First, nausea was considered present if additional antiemetics were prescribed. Since this is retrospective observational study, data on the visual analog scale and numerical rating scale, which are used to evaluate nausea, are missing. Furthermore, previous studies have evaluated chemotherapy-induced nausea and vomiting, and reported that the evaluation of nausea differed between patients and physicians.^20,21^

Second, few opioid-naïve patients received opioid injections as the initial opioid treatment. Thus, opioid-naïve patients in the present study may have acquired drug resistance to opioids through their previous experiences of oral or patch opioids. A large trial that takes conditions of patients with end-stage cancer into account will be needed.

## Figures and Tables

**Table1:** Characteristics of Prochlorperazine and Placebo

	Prochlorperazine n=103	Placebo n=53	P-value
Sex (Male/Male)	51/52	29/24	0.538^1)^
age	78 (45–98)	76 (50–99)	0.333^2)^
Performance Status^1)^			
1	0	0	
2	10	10	0.130
3	43	18	0.389
4	50	25	1.000
Carcinoma^1)^			
Esophagus and Stomach	13	6	1.000
Colon	9	2	0.335
Liver, Pancreas, Bile duct	22	10	0.853
Lung	25	10	0.545
Hematopoietic tumors	3	1	1.000
Brest	0	2	0.114
Gynecology	7	5	0.543
Urology	11	7	0.792
Head and neck	9	5	1.000
Unknown	1	1	1.000
Skin	0	3	0.039^※^
Sarcoma	3	0	0.551
Thymus cancer	0	1	0.335

1) Fisher’s t-test 2) Mann-Whitney U test * P<0.05, ** P<0.01

**Table2:** The opioid types before and after opioid switching

Prochlorperazine (n=103)				
	Injection (After Opioid Switching)			Total
	Morphine	Oxycodone	Hydromorphone	
Before Opioid Switching				
Morphine	14	1	0	15
Oxycodone	16	11	1	28
Hydromorphone	0	1	0	1
Fentanyl tape	5	3	0	8
None (Naive)	34	16	1	51

	69	32	2	

Placebo (n=53)				
	Injection (After Opioid Switching)			Total
	Morphine	Oxycodone	Hydromorphone	
(Before Opioid Switching)				
Morphine	9	0	0	9
Oxycodone	6	18	0	24
Hydromorphone	0	1	1	2
Fentanyl tape	5	3	0	8
None (Naive)	6	4	0	10

	26	26	1	

**Table3:** Opoiod naive patients in the Prochlorperazine and Placebo

None (Naive)			
	Prochlorperazine n=103	Placebo n=53	P-value
Opioid Injections	51(49.5)	10(18.9)	<0.01**

Fisher’s t-test ** P<0.01

**Table4:** Incidence of OINV in Prochlorperazine and Placebo

	Prochlorperazine n=103	Placebo n=53	Odds Ratio	95% CI
OINV	4（3.9％）	1（1.9％）	2.101	0.305–14.259

**Table5:** Prochlorperazine and drowsiness

	Prochlorperazine n=103	Placebo n=53	P-value
Sleepiness	4 (1)	12 (8)	0.193
Sleepless	99 (13)	41 (8)	
Use of sleeping medicine for sleepless	9/13	2/8	

( ): Sleepiness provoking factors Fisher’s t-test

## Supplementary Material

PDF-Japanese

## References

[1] Fischer DJ, Villines D, Kim YO, Epstein JB, Wilkie DJ. Anxiety, depression, and pain differences by primary cancer. Support Care Cancer 2010; 18: 801–810. 19685346 10.1007/s00520-009-0712-5 PMC3682075

[2] Bruera E, Kim HN. Cancer pain. JAMA 2003; 290: 2476–2479. 14612485 10.1001/jama.290.18.2476

[3] Davis MP, Walsh D. Epidemiology of cancer pain and factors influencing poor pain control. Am J Hosp Palliat Care 2004; 21: 137–142. 15055515 10.1177/104990910402100213

[4] Wiffen PJ, Derry S, Moore RA. Impact of morphine, fentanyl, oxycodone or codeine on patient consciousness, appetite and thirst when used to treat cancer pain. Cochrane Database Syst Rev 2014; 2014: CD011056. 24874470 10.1002/14651858.CD011056.pub2 PMC6483540

[5] Mercadante S, Bruera E. Opioid switching: a systematic and critical review. Cancer Treat Rev 2006; 32: 304–315. 16624490 10.1016/j.ctrv.2006.03.001

[6] Quigley C. Opioid switching to improve pain relief and drug tolerability. Cochrane Database Syst Rev 2013; (10): CD004847. 24142865 10.1002/14651858.CD004847.pub2 PMC10658828

[7] Enting RH, Oldenmenger WH, van der Rijt CC, Wilms EB, Elfrink EJ, Elswijk I, Sillevis Smitt PA. A prospective study evaluating the response of patients with unrelieved cancer pain to parenteral opioids. Cancer 2002; 94: 3049–3056. 12115396 10.1002/cncr.10518

[8] Naeim A, Dy SM, Lorenz KA, Sanati H, Walling A, Asch SM. Evidence-based recommendations for cancer nausea and vomiting. J Clin Oncol 2008; 26: 3903–3910. 18688059 10.1200/JCO.2007.15.9533

[9] Laugsand EA, Kaasa S, Klepstad P. Management of opioid-induced nausea and vomiting in cancer patients: systematic review and evidence-based recommendations. Palliat Med 2011; 25: 442–453. 21708851 10.1177/0269216311404273

[10] McNicol E, Horowicz-Mehler N, Fisk RA, Bennett K, Gialeli-Goudas M, Chew PW, Lau J, Carr D. Management of opioid side effects in cancer-related and chronic noncancer pain: a systematic review. J Pain 2003; 4: 231–256. 14622694 10.1016/s1526-5900(03)00556-x

[11] Glare P, Pereira G, Kristjanson LJ, Stockler M, Tattersall M. Systematic review of the efficacy of antiemetics in the treatment of nausea in patients with far-advanced cancer. Support Care Cancer 2004; 12: 432–440. 15108099 10.1007/s00520-004-0629-y

[12] National Comprehensive Cancer Network. Management of opioid adverse effects. In: NCCN clinical practice guidelines in oncology. Adult cancer pain. Version 1; 2018. <https://oncolife.com.ua/doc/nccn/Adult_Cancer_Pain.pdf January 22> (Accessed 01 February, 2020)

[13] Walsh D, Davis M, Ripamonti C, Bruera E, Davies A, Molassiotis A. 2016 Updated MASCC/ESMO consensus recommendations: Management of nausea and vomiting in advanced cancer. Support Care Cancer 2017; 25: 333–340. 27534961 10.1007/s00520-016-3371-3

[14] Tsukuura H, Miyazaki M, Morita T, Sugishita M, Kato H, Murasaki Y, Gyawali B, Kubo Y, Ando M, Kondo M, Yamada K, Hasegawa Y, Ando Y. Efficacy of prophylactic treatment for oxycodone-induced nausea and vomiting among patients with cancer pain (POINT): A randomized, placebo-controlled, double-blind trial. Oncologist 2018; 23: 367–374. 29038236 10.1634/theoncologist.2017-0225 PMC5905679

[15] Good P, Richard R, Syrmis W, Jenkins-Marsh S, Stephens J. Medically assisted hydration for adult palliative care patients (Review). Cochrane Database Syst Rev 2014; (4): CD006273. 24760678 10.1002/14651858.CD006273.pub3 PMC8988261

[16] Anderson F, Downing GM, Hill J, Casorso L, Lerch N. Palliative performance scales (PPS): a new tool. J Palliat Care 1996; 12: 5–11. 8857241

[17] Stephenson J, Davies A. An assessment of aetiology-based guidelines for the management of nausea and vomiting in patients with advanced cancer. Support Care Cancer 2006; 14: 348–353. 16228185 10.1007/s00520-005-0897-1

[18] Ng K, von Gunten CF. Symptoms and attitudes of 100 consecutive patients admitted to an acute hospice/ palliative care unit. J Pain Symptom Manag 1998; 16: 307–316. 10.1016/s0885-3924(98)00097-9 9846025

[19] Hugel H, Ellershaw JE, Cook L, Skinner J, Irvine C. The prevalence, key causes and management of insomnia in palliative care patients. J Pain Symptom Manage 2004; 27: 316–321. 15050659 10.1016/j.jpainsymman.2003.09.010

[20] Yamamoto Y, Tsukiyama I, Inuzuka R, Yabushita H, Wakatsuki A, Matsuura K. A substantial investigation of discrepancy between patient complaints and assessment by medical personnel in chemotherapy—induced nausea. Palliat Care Res 2015; 10: 142–148.

[21] Vidall C, Fernández-Ortega P, Cortinovis D, Jahn P, Amlani B, Scotté F. Impact and management of chemotherapy/radiotherapy-induced nausea and vomiting and the perceptual gap between oncologists/oncology nurses and patients: a cross-sectional multinational survey. Support Care Cancer 2015; 23: 3297–3305. 25953380 10.1007/s00520-015-2750-5 PMC4584113

