# Supplementary material for: The efficacy of prophylactic prochlorperazine injections at the initiation of opioid injections in preventing opioid-induced nausea and vomiting among patients with end-stage cancer
Source: Fujita Med J. 2023 Aug 28;9(4):270–4. doi: 10.20407/fmj.2022-034 (PMC10701219; doi:10.20407/fmj.2022-034)

終末期癌患者に対するオピオイド注射薬導入時におけるプロクロルペラジン注射薬によるオピオイド誘発性悪心嘔吐の予防効果に関する検討

Kazuki Imai[1-3], Akihiko Futamura, PhD[2,3], Miyo Murai,MD[2], PhD, Akihiro Ito, MD, PhD[2], Norimasa Tsuzuki, MD[2], Masanobu Usui, MD, PhD[2]

1.Fujita Health University Hospital, Toyoake, Aichi, Japan

2. Department of Surgery and Palliative Medicine, Fujita Health University, School of Medicine, Toyoake, Aichi, Japan

3. Department of Pharmacy, Fujita Health University Nanakuri Memorial Hospital, Tsu, Mie, Japan

Original Article

ランニングタイトル: オピオイド誘発性悪心嘔吐予防に対するプロクロルペラジン注射薬の有用性

Corresponding author: Kazuki Imai

Department of Department of Pharmacy, Fujita Health University Nanakuri Memorial Hospital, 424-1, Oodori-cho, Tsu, Mie 514-1295, Japan059-252-1555

k-imai＠fujita-hu.ac.jp

【アブストラクト】

Objectives:オピオイド誘発性悪心嘔吐 OINV（Opioid Induce Nausea and Vomiting ; OINV）発症時の制吐薬投与は広く推奨されている。一方で、OINV の予防的投与に関する経口オキシコドン導入時のプロクロルペラジンの臨床試験では、その有用性は示されなかったと報告がある。そこで、既報とは異なる終末期がん患者において注射用のプロクロルペラジンが使用された患者を対象に、OINV の予防効果および眠気誘発について検討した。

Methods: 2017 年 4 月から 2020 年 3 月に、オピオイド開始から 5 日以上、オピオイ

ド注射薬で治療された患者を対象に、プロクロルペラジン注射薬を併用した患者とオピオイド単剤で治療された患者を2群に分けて、プロクロルペラジン投与のOINVに対する予防効果およびプロクロルペラジン注による眠気誘発に関して調査した。終末期がん患者の全身状態の指標はPerformance Status（PS）のほか、予後予測指標であるPalliative Performance Scale（PPS）も用いた。

Results：対象は325例中156例が該当した。併用投与が103例、単独投与が53例。両群の患者背景、年齢・性別・PS・PPSに有意差なし。OINVの発生は併用群で4例、単独群で1例であった。プロクロルペラジン注による眠気誘発を検討したが、病状より睡眠障害を併発することも多くプロクロルペラジン注が起因であるとは一概に言えない結果となった。

Conclusions：オピオイド注射薬で治療している患者においても既報と同様に、プロクロルペラジ注射薬の予防効果は認められなかった。

Keyword: OINV、モルヒネ、オキシコドン、プロクロルペラジン注射薬

【Introduction】

疼痛は癌患者の20～50％で生じ[1]、進行癌患者では約80％が中等度から重度の疼痛を生じ[2]、オピオイド鎮痛薬を中心とした薬物療法が基本となる。World Health Organization（WHO）の3段階除痛ラダーは、疼痛強度を重症度に従って非オピオイド鎮痛薬、オピオイド鎮痛薬をその強さに基づいて推奨するものである。[3]

オピオイド鎮痛薬によるOINV（Opioid Induce Nausea and Vomiting；OINV）の副作用は、患者のQOLを著しく低下させるため、その予防が極めて重要である。実際に経口または貼付オピオイド鎮痛薬(Oral or Patch opioid)開始により約20%に悪心が、約10%に嘔吐が発現するとの報告がある[4]。これまで、OINV発症時は経口薬から注射薬への変更、異系統への変更、制吐薬の使用により症状を改善することが示唆されている。[5-11]

しかし、OINVに対する予防投与に関して、National Comprehensive Cancer Network

（NCCN）[12]やMultinational Association of Supportive Care in Cancer (MASCC) とThe European Society for Medical Oncology (ESMO)[13]などのガイドラインにおいて明確に言及されていない。

Tsukuuraらは、経口オキシコドン導入時のOINVに対するプロクロルペラジンの予防投与に関する臨床試験において、有用性を示さなかったと報告している[14]。

既報の患者背景はEastern Cooperative Oncology Group のPerformance Status（以下、PS）が2以下の患者が全体で120例中92例（76.7）％を占めていた。しかし、癌終末期においては、悪液質の進展に伴い食思不振、全身衰弱、悪心、意識レベルの低下などが出現し、経口投与が困難[15]となり、注射薬を使用せざるを得ないことが多くある。そして、癌終末期となるとPSも既報とは異なり3以上の患者が多く占めることが予測されるため、PSに加え、緩和ケア領域で予後予測指標として知られているPalliative Performance Scale(PPS)[16]を用いて、予後3か月未満の終末期の患者

を対象に調査した。また、既報ではプロクロルペラジンの有用性は認められず、副作用である眠気が多く報告されたため眠気に関しても病期で異なるかを調査した。そこで、主要評価項目はオピオイド注射薬にプロクロルペラジン注射薬を併用した群とオピオイド注射薬単剤群を比較し、プロクロルペラジン注射薬による制吐作用の有効性を明らかにすることとした。また、副次評価項目はプロクロルペラジン注射薬による眠気の誘発とした。

【Methods】

1.対象患者

対象患者は、2017 年 4 月から 2020 年 3 月までに藤田医科大学七栗記念病院の緩和ケア病棟に入院した終末期がん患者 325 例を対象とし、オピオイド注射薬の開始と同時に 5 日間制吐薬としてプロクロルペラジン注射薬 5mg が投与された群（プロクロルペラジン群）と、オピオイド注射薬単独投与群（プラセボ群）に分けて電子カルテシ

ステムから後方視的に調査を行った。

2. 評価方法

対象患者の適格基準は、図 1 に示す通りオピオイド注投与開始日から 7 日以上の生存期間を有し、オピオイド注射薬単独投与群は開始から連続 5 日間とし、プロクロルペラジン群は両剤開始日からプロクロルペラジン注射薬を 5 日間連続投与している場合とした。

悪心嘔吐の判定は、プロクロルペラジン注射薬および他の制吐薬の追加投与が必要であった場合とした。

除外基準は、次の通りとした。

１.適格基準を満たさない、2.オピオイド注射薬投与前から嘔気が存在 、3.他の制吐薬（ハロペリドール、メトクロプラミド、オクトレオチド、クロミフェラミン、ステロイド）の使用、4 .意思疎通が不可能、5.脳転移、6.高カルシウム血症とした。

3.統計解析

両群間における量的データ比較には Mann-Whitney U test を用い、質的データの比較には Fisher’s t-test を用い、$p < 0.05$ を統計学的有意水準とした。プロクロルペラジン群とプラセボ群における悪心嘔吐の発現および性差による悪心嘔吐には Odds Ratio を用いた。統計解析には、Statcel 3 （4 Steps Excel-Statistics、OMS Publishing Ltd、Saitama、Japan） を用いた。

4.倫理規定

本研究は「人を対象とする医学系研究に関する倫理指針」を順守し、藤田医科大学倫理審査委員会の承認（倫理委員会受付番号 ： CI20-641） を得て実施した。

【Results】

調査期間内にオピオイド注射薬が開始となった患者は 325 例であった。そのうち、評価の適格基準を満たさない 169 例が除外され、156 名を対象に調査した。

除外対象となった内訳は、生存期間が評価期間を満たさなかった患者は20例。　オピオイド注射薬投与前から悪心が存在した患者は25例。せん妄によるハロペリドール注射薬を使用した患者が56例。プロクロルペラジン注射薬以外の制吐薬を使用した患者は39例。そのうちの8例は腹膜播種や消化管閉塞によりオクトレオチド注が投与されていた。意思疎通が不可能な患者は14例。評価期間中のオピオイド注射薬またはプロクロルペラジン注射薬使用回数不足患者は10例。入院時よりオピオイド注射薬投与患者は3例。脳転移と高カルシウム血症などの悪心嘔吐誘発因子を有する患者はそれぞれ1例であった。

対象患者156名のうちプロクロルペラジン注射薬投与患者（プロクロルペラジン群）は103例（66.0%）、非投与患者（プラセボ群）は53例（34.0%）となった。（図2）

プロクロルペラジン群とプラセボ群において、性別 、年齢、Eastern Cooperative Oncology Group Performance Status (PS)、に有意な差は認められなかった。また、

がん種別でも、プロクロルペラジン群とプラセボ群間で差は認められなかった。(Table 1)また、PPS においても有意な差は認められなかった。(Figure 3)

　プロクロルペラジン群とプラセボ群のオピオイドスイッチング前後における使用オピオイドを調査した（Table2)。

　その結果、プロクルペラジン群 103 例中、オピオイド注射薬導入する以前のオピオイド薬は、投与頻度が高い順にオキシコドン経口薬が 28 例（27.2%)、モルヒネ経口薬が 15 例（14.6%)、フェンタニル貼付薬が 8 例（7.8%)、ヒドロモルフォン経口薬が 1 例(1.0%)であり､投与歴がなかった患者(オピオイドナイーブ患者)は 51 例(49.5%)であった。

　オピオイドスイッチングによるオピオイド注射薬の投与頻度が高い順位に、モルヒネ注射薬 69 例（67.0%)、オキシコドン注射薬が 32 例（31.1%)、ヒドロモルフォン注射薬が 2 例（1.9%）であった。

オピオイドスイッチングによるオピオイド薬の種類の変化は、モルヒネ経口薬 15 例からモルヒネ注射薬が 14 例（93.3%）、オキシコドン注射薬が 1 例（6.7%）。オキシコドン経口薬 28 例から、モルヒネ注射薬が 16 例（60.7%）、オキシコドン注射薬が 11 例（39.3%）、ヒドロモルフォン注射薬は 1 例（3.6%）。1 例のヒドロモフフォン経口薬はオキシコドン注射薬へ。フェンタニル貼付薬 8 例からモルヒネ注射薬が 5 例(62.5%)、オキシコドン注射薬が 3 例（37.5%）。オピオイドナイーブ患者 51 例では、モルヒネ注射薬が 34 例（66.7%）、オキシコドン注射薬が 16 例（31.4%）、ヒドロモルフォン注射薬が 1 例（2.0%）で開始となった。

プラセボ群 53 例中、オイド注射薬導入する以前のオピオイド薬は、投与頻度が高い順にオキシコドン経口薬が 24 例（45.3%）、モルヒネ経口薬が 9 例（17.0%）、フェンタニル貼付薬が 8 例（15.9%）、ヒドロモルフォン経口薬が 2 例（3.8%）でありオピオイドナイーブ患者は 10 例（18.9%）であった。

オピオイドスイッチングによるオピオイド注射薬の投与頻度が高い順位に、モルヒネ注射薬とオキシコドン注射薬がともに 26 例（49.1%)、ヒドロモフフォン注射薬が 1 例（1.8%）であった。

オピオイドスイッチングによるオピオイド薬の種類の変化は、モルヒネ経口薬 9 例は全例でモルヒネ注射薬へ。オキシコドン経口薬 24 例からモルヒネ注射薬が 6 例（25.0%)、オキシコドン注射薬が 18 例（75.0%)。ヒドロモフォン経口薬 2 例からオキシコドン注射薬とヒドロモルフォン注射薬がそれぞれ 1 例ずつであった。フェンタニル貼付薬 8 例からモルヒネ注射薬が 5 例（62.5%)、オキシコドン注射薬が 3 例（37.5%)。オピオイドナイーブ患者 10 例では、モルヒネ注射薬が 6 例（60.0%)、オキシコドン注射薬が 4 例（40.0%）であった。(Table 2)。

また、オピオイドナイーブ投与患者 61 例に限り調査した結果、プロクロルペラジン群が 51 例（49.5%)、プラセボ群が 10 例（18.9%）であった。(p=0.0002)（Table3）

オピオイド注射薬開始 5 日間の OINV の発生は、プロクロルペラジン群では 103 例中 4 例 (3.9%) 、プラセボ群は 53 例中 1 例 (1.9%) で有意差は認められなかった (p=0.502)。(Table 4)

眠気はプロクロルペラジン群で 4 例、プラセボ群で 12 例であった。プロクロルペラジン群 4 例のうち 1 例 (25.0%) で眠剤の使用と増量があり、プラセボ群 12 例のうち 6 例が眠剤を使用し 2 例はオピオイドの増量があった (66.7%)。

眠気の誘発はなかったが睡眠導入薬や抗うつ薬などの催眠誘発薬を使用した患者は、プロクロルペラジン群で 13 例、プラセボ群で 8 例であった。そのうちプロクロルペラジン群 9 例 (69.2%)、プラセボ群 2 例 (25.0%) で、不眠を訴え催眠薬が投与された。

催眠誘発因子を除いた例数でプロクロルペラジン注射薬と考えられる眠気の検討を行った結果、有意差は認められなかった。(P=0.193) (Table.5)

【Discussion】

今回の調査結果から、先行研究と異なる癌終末期およびプロクロルペラジンの剤型の違いにおいても OINV への制吐効果は乏しいことが示唆された。

プロクロルペラジン群およびプラセボ群において、いずれもスイッチ前に経口オキシコドン薬が最も多く選択されており、スイッチ後はモルヒネ注射薬へのスイッチが増加していた。内服が困難な状態に進行したがん患者の多くは、痛みのほか、呼吸困難感、倦怠感などの苦痛症状が発現してくるため、医師の臨床経験からモルヒネ注射薬を選択している傾向を認めた。このことから、プロクロルペラジン群はプラセボ群と比較してオピオイドスイッチングにより異なる種類のオピオイド薬を選択する場合やオピオイドナイーブ患者に対して OINV 発症予防に投与されていると考えられた (Table3)。さらに、オピオイドナイーブ患者に限定し検討した結果 (Table4)、プロクロルペラジン群とプラセボ群において OINV のオッズ比よりプロ

クロルペラジン注射薬を支持する結果は示されなかった。

今回の結果においては、プロクロルペラジン群の OINV 発現はプラセボ群と比べ高くなった。予防投与および OINV に限定した報告ではないが、悪心嘔吐に対し有効である可能性があるとした既報[10, 11]と比較すると異なる結果となった。プロクロルペラジン群とプラセボ群を合わせた全患者を対象にした場合、悪心嘔吐発現数は 5 例（発現率：3.2%）であった。これは、オピオイド経口薬・貼付薬開始時に発現する悪心嘔吐割合よりも低い結果となった[4]。この結果には、2 つの可能性が考えられる。1 つ目は Stephenson ら[17]により報告された耐性の獲得と、2 つ目は Enting ら[7]らにより報告されたオピオイド経口薬からオピオイド注射薬へのオピオイドスイッチングまたは導入が考えられる。今回の結果においてもオピオイドに対する耐性を形成していた可能性、または、オピオイド経口薬・貼付薬と比べ悪心嘔吐発現が少ないオピオイド注射薬を使用したためと思われる。

副次評価項目としてプロクロルペラジン注射薬による眠気を検討したが、プロクロルペラジン群とプラセボ群においてプロクロルペラジン注射薬と眠気の因果関係は認められず、 Tsukuura ら[14]との報告と異なった。これは、今回対象となった終末期癌患者の 23%から 70%の頻度で眠気は発生するといわれており[18, 19]、患者層の違いであると考えられる。

今回の結果より、終末期がん患者における OINV に対するプロクロルペラジン注射薬予防投与は、有効性および眠気誘発性に有意差は示されなかった。したがって、オピオイド導入に際し、一律に制吐薬を処方することないと考えられる。ただし、悪心嘔吐の要因には、性別、癌の進展、中枢性であるか末梢性など、様々な要因を絡んでいるため、それら要因と症状に合わせた適切な制吐薬の使用が望まれる。

本研究には限界がある。１つは、悪心の判断は追加制吐薬の使用の有無を基準としたことである。なぜなら、本研究は後ろ向き観察研究であり悪心への評価尺度である

Visual Analogue Scale(VAS)やNumerical Rating Scale(NRS)の欠損が認められたこと、また、化学療法誘発性悪心嘔吐ではあるが悪心に対する評価は患者と医療者で乖離している報告もあるためである[20,21]。

2つ目は、オピオイドナイーブ患者へオピオイド注射薬が導入として投与されることが少ないため、注射薬前の経口または貼付オピオイド製剤により耐性が形成されている可能性がある。したがって、今後は、終末期癌患者の病態を踏まえての大規模な調査も検討課題として必要と考える。

【Acknowledgements】

本論文の要旨は日本病院薬剤師会東海ブロック・日本薬学会東海支部合同学術集会2021で発表した。

開示すべき利益相反はない。

参考文献

1)Fischer DJ, Villines D, Kim YO, Epstein JB, Wilkie DJ.. Anxiety, depression, and pain differences by primary cancer. Support Care Cancer 2010; 18: 801-10.

2)Bruera E, Kim HN. Cancer pain. JAMA 2003 ; 290: 2476-9.

3) Davis MP, Walsh D. Epidemiology of cancer pain and factors influencing poor pain control. Am J Hosp Palliat Care 2004; 21: 137-42.

4) Wiffen PJ, Derry S, Moore RA. Impact of morphine, fentanyl, oxycodone or codeine on patient consciousness, appetite and thirst when used to treat cancer pain. Cochrane DatabaseSyst Rev 2014; 2014: CD011056.

5)Mercadante S, Bruera E. Opioid switching: a systematic and critical review. Cancer Treat Rev 2006; 32: 304-15.

6) Quigley C. Opioid switching to improve pain relief and drug tolerability. Cochrane Database Syst Rev 2013； (10)： CD004847.

7) Enting RH, Oldenmenger WH, van der Rijt CC, Wilms EB, Elfrink EJ, Elswijk I, Sillevis Smitt PA. A prospective study evaluating the response of patients with unrelieved cancer pain to parenteral opioids. Cancer 2002； 94： 3049-56.

8)Naeim A, Dy SM, Lorenz KA, Sanati H, Walling A, Asch SM. Evidence-based recommendations for cancer nausea and vomiting. J Clinical Oncology 2008 ； 26： 3903-10.

9) Laugsand EA, Kaasa S, Klepstad P. Management of opioid-induced nausea and vomiting in cancer patients： systematic review and evidence-based recommendations. Palliat Med 2011； 25： 442-53.

10) McNicol E, Horowicz-Mehler N, Fisk RA, Bennett K, Gialeli-Goudas M, Chew PW, Lau J, Carr D. Management of opioid side effects in cancer-related and chronic noncancer pain: a systematic review. J Pain 2003; 4: 231-56.

11) Glare P, Pereira G, Kristjanson LJ, Stockler M, Tattersall M. Systematic review of the efficacy of antiemetics in the treatment of nausea in patients with far-advanced cancer. Support Care Cancer 2004; 12: 432-40.

12) National comprehensive cancer network. Management of opioid adverse effects. In: NCCN clinical practice guidelines in oncology. Adult cancer pain. Version 1;2018. https://oncolife.com.ua/doc/nccn/Adult_Cancer_Pain.pdf January 22, 2018

13) Walsh D, Davis M, Ripamonti C, Bruera E, Davies A, Molassiotis A. 2016 Updated MASCC/ESMO consensus recommendations: Management of nausea and

vomiting in advanced cancer. Support Care Cancer 2017 ; 25： 333-40.

14) Tsukuura H, Miyazaki M, Morita T, Sugishita M, Kato H, Murasaki Y, Gyawali B, Kubo Y, Ando M, Kondo M, Yamada K, Hasegawa Y, Ando Y. Efficacy of Prophylactic Treatment for Oxycodone-Induced Nausea and Vomiting Among Patients with Cancer Pain (POINT)： A Randomized, Placebo-Controlled, Double-Blind Trial. Oncologist 2018; 23： 367-74.

15) Good P, Richard R, Syrmis W, Jenkins-Marsh S, Stephens J. Medically assisted hydration for adult palliative care patients (Review). Cochrane Database Syst Rev 2014; (4)： CD006273.

16) Anderson F, Downing GM, Hill J： Palliative performance scales (PPS) ： a new tool. J Palliative Care 1996; 12： 5-11

17) Stephenson J, Davies A. An assessment of aetiology-based guidelines for

the management of nausea and vomiting in patients with advanced cancer. Support Care Cancer 2006；14： 348-3.

18) Ng K, von Gunten CF. Symptoms and attitudes of 100 consecutive patients admitted to an acute hospice/ palliative care unit. J Pain Symptom Manage 1998；16：307–16.

19) Heino Hugel, John E Ellershaw, Lucy Cook, Jennifer Skinner, Caroline Irvine. The prevalence, key causes and management of insomnia in palliative care patients. J Pain Symptom Manage 2004；27：316–21.

20) Yamamoto Y, Tsukiyama I, Inuzuka R, Yabushita H, Wakatsuki A, Matsuura K. A substantial investigation of discrepancy between patient complaints and assessment by medical personnel in chemotherapy–induced nausea. Palliative Care Research 2015；10：142-8.

21) Vidall C, Fernández-Ortega P, Cortinovis D, Jahn P, Amlani B, Scotté F. Impact and management of chemotherapy/radiotherapy-induced nausea and vomiting and the perceptual gap between oncologists/oncology nurses and patients: a cross-sectional multinational survey. Support Care Cancer 2015; 23: 3297-305.

Table 1

	Prochlorperazine n = 103	Placebo n = 53	P-value
Sex(Male/Male)	51/52	29/24	0.538[1)]
age	78(45-98)	76(50-99)	0.333[2)]
Performance Status [1)]			
1	0	0	
2	10	10	0.130
3	43	18	0.389
4	50	25	1.000
Carcinoma [1)]			
Esophagus and Stomach	13	6	1.000
Colon	9	2	0.335
Liver, Pancreas, Bile duct	22	10	0.853
Lung	25	10	0.545
Hematopoietic tumors	3	1	1.000
Brest	0	2	0.114
Gynecology	7	5	0.543
Urology	11	7	0.792
Head and neck	9	5	1.000
Unknown	1	1	1.000
Skin	0	3	0.039[※]
Sarcoma	3	0	0.551
Thymus cancer	0	1	0.335

1) Fisher's t-test

2) Mann-Whitney U test

*P < 0.05, **P < 0.01

Table 2

		Prochlorperazine (n = 103)			
		Injection (After Opioid Switching)			
		Morphine	Oxycodone	Hydromorphone	
Before Opioid Switching	Morphine	14	1	0	15
	Oxycodone	16	11	1	28
	Hydromorphone	0	1	0	1
	Fentanyl tape	5	3	0	8
	None (Naive)	34	16	1	51
		69	32	2	

		Placebo (n = 53)			
		Injection (After Opioid Switching)			
		Morphine	Oxycodone	Hydromorphone	
(Before Opioid Switching)	Morphine	9	0	0	9
	Oxycodone	6	18	0	24
	Hydromorphone	0	1	1	2
	Fentanyl tape	5	3	0	8
	None (Naive)	6	4	0	10
		26	26	1	

Table 3

	None (Naive)		
	Prochlorperazine n = 103	Placebo n = 53	P-value
Opioid Injections	51(49.5)	10(18.9)	< 0.01**

Fisher's t-test

**P < 0.01

Table 4

Incidence of OINV in Prochlorperazine and Placebo

	Prochlorperazine n = 103	Placebo n = 53	Odds Ratio	95% CI
OINV	4 (3.9%)	1 (1.9%)	2.101	0.305 - 14.259

Table 5

	Prochlorperazine n = 103	Placebo n = 53	P-value
Sleepiness	4 (1)	12 (8)	0.193
Sleepless	99 (13)	41 (8)	
Use of sleeping medicine for sleepless	9/13	2/8	

( ) : Sleepiness provoking factors

Fisher's t-test

Figure 1

Eligibility criteria

Evaluation period	Day1	Day2	Day3	Day4	Day5	Day6	Day7
Survival Time	●	●	●	●	●	●	●
Prochlorperazine	●	●	●	●	●		
or							
Placebo	●	●	●	●	●		

Figure 2

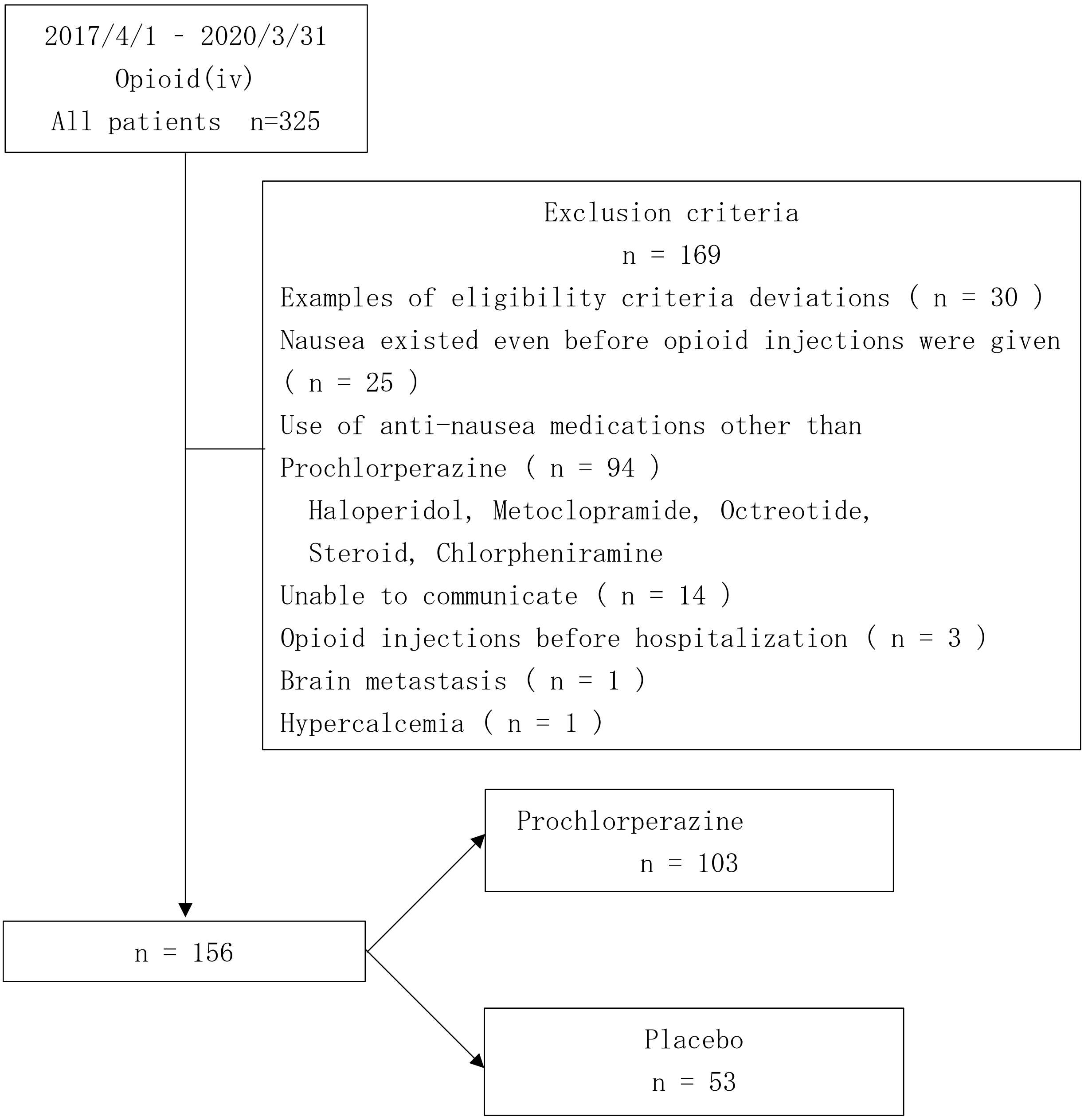
2017/4/1 - 2020/3/31
Opioid(iv)
All patients n=325
Exclusion criteria
n = 169
Examples of eligibility criteria deviations (n = 30)
Nausea existed even before opioid injections were given (n = 25)
Use of anti-nausea medications other than Prochlorperazine (n = 94)
Haloperidol, Metoclopramide, Octreotide, Steroid, Chlorpheniramine
Unable to communicate (n = 14)
Opioid injections before hospitalization (n = 3)
Brain metastasis (n = 1)
Hypercalcemia (n = 1)
n = 156
Prochlorperazine
n = 103
Placebo
n = 53

Figure.3
Palliative Performance Scale

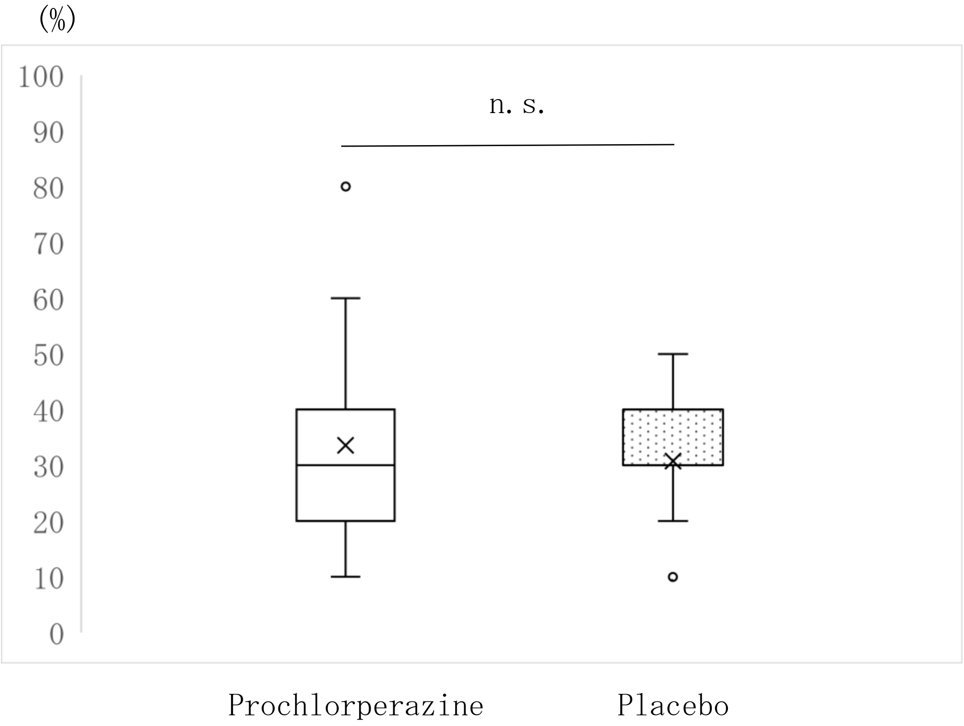

Supplement: Supplementary file 1 — PDF-Japanese [file fmj-9-270-s001.pdf]
